# Supplementary material for: Mental well-being in Swedish adolescents 2014–2023: A repeated population-based cross-sectional study focusing on temporal variations and differences between groups
Source: PLoS One. 2025 May 28;20(5):e0323963. doi: 10.1371/journal.pone.0323963 (PMC12118832; doi:10.1371/journal.pone.0323963)
Supplement: S1 Table — (DOCX) [file pone.0323963.s001.docx]

| Coefficients^a^ | | | | | | | | |
| --- | --- | --- | --- | --- | --- | --- | --- | --- |
| Model | | Unstandardized Coefficients | | Standardized Coefficients |  | 95,0% Confidence Interval for B | | Collinearity Statistics |
|  |  | B | Std. Error | Beta | Sig. | Lower Bound | Upper Bound | VIF |
| 1 | (Constant) | 35,836 | 0,394 |  | 0,000 | 35,064 | 36,608 |  |
|  | Gender | 4,916 | 0,250 | 0,173 | 0,000 | 4,426 | 5,407 | 1,000 |
| 2 | (Constant) | 30,628 | 0,497 |  | 0,000 | 29,653 | 31,603 |  |
|  | Gender | 4,458 | 0,249 | 0,157 | 0,000 | 3,970 | 4,946 | 1,012 |
|  | Sexual orientation | 6,637 | 0,394 | 0,147 | 0,000 | 5,864 | 7,410 | 1,012 |
| 3 | (Constant) | 31,354 | 0,497 |  | 0,000 | 30,380 | 32,327 |  |
|  | Gender | 4,632 | 0,247 | 0,163 | 0,000 | 4,147 | 5,117 | 1,015 |
|  | Sexual orientation | 6,146 | 0,393 | 0,136 | 0,000 | 5,376 | 6,917 | 1,021 |
|  | NDD | -5,759 | 0,420 | -0,119 | 0,000 | -6,582 | -4,937 | 1,010 |
| 4 | (Constant) | 31,466 | 0,497 |  | 0,000 | 30,492 | 32,440 |  |
|  | Gender | 4,652 | 0,247 | 0,163 | 0,000 | 4,167 | 5,137 | 1,015 |
|  | Sexual orientation | 6,090 | 0,393 | 0,135 | 0,000 | 5,320 | 6,861 | 1,022 |
|  | NDD | -5,504 | 0,424 | -0,114 | 0,000 | -6,335 | -4,674 | 1,031 |
|  | Hearing impairment | -2,479 | 0,587 | -0,037 | 0,000 | -3,631 | -1,328 | 1,023 |
| 5 | (Constant) | 22,182 | 0,619 |  | 0,000 | 20,968 | 23,397 |  |
|  | Gender | 4,252 | 0,242 | 0,149 | 0,000 | 3,777 | 4,727 | 1,020 |
|  | Sexual orientation | 5,579 | 0,385 | 0,124 | 0,000 | 4,825 | 6,333 | 1,025 |
|  | NDD | -4,854 | 0,415 | -0,100 | 0,000 | -5,668 | -4,040 | 1,035 |
|  | Hearing impairment | -2,211 | 0,574 | -0,033 | 0,000 | -3,337 | -1,085 | 1,024 |
|  | SSES | 2,842 | 0,118 | 0,205 | 0,000 | 2,611 | 3,073 | 1,014 |
| 6 | (Constant) | 23,064 | 0,682 |  | 0,000 | 21,728 | 24,401 |  |
|  | Gender | 4,272 | 0,242 | 0,150 | 0,000 | 3,797 | 4,747 | 1,021 |
|  | Sexual orientation | 5,533 | 0,385 | 0,123 | 0,000 | 4,778 | 6,287 | 1,026 |
|  | NDD | -4,766 | 0,416 | -0,098 | 0,000 | -5,581 | -3,951 | 1,040 |
|  | Hearing impairment | -2,367 | 0,576 | -0,035 | 0,000 | -3,497 | -1,237 | 1,032 |
|  | SSES | 2,827 | 0,118 | 0,204 | 0,000 | 2,596 | 3,057 | 1,016 |
|  | Survey year | -0,320 | 0,104 | -0,026 | 0,002 | -0,523 | -0,117 | 1,015 |
| 7 | (Constant) | 26,039 | 1,042 |  | 0,000 | 23,997 | 28,081 |  |
|  | Gender | 2,198 | 0,575 | 0,077 | 0,000 | 1,071 | 3,326 | 5,761 |
|  | Sexual orientation | 5,477 | 0,385 | 0,121 | 0,000 | 4,722 | 6,231 | 1,028 |
|  | NDD | -4,767 | 0,416 | -0,098 | 0,000 | -5,582 | -3,952 | 1,040 |
|  | Hearing impairment | 0,546 | 1,268 | 0,008 | 0,667 | -1,940 | 3,032 | 5,003 |
|  | SSES | 2,831 | 0,118 | 0,204 | 0,000 | 2,601 | 3,062 | 1,016 |
|  | Survey year | -1,487 | 0,324 | -0,122 | 0,000 | -2,122 | -0,852 | 9,965 |
|  | KonxLar | 0,819 | 0,206 | 0,130 | 0,000 | 0,416 | 1,222 | 14,917 |
|  | HorxLar | -1,350 | 0,522 | -0,048 | 0,010 | -2,373 | -0,327 | 4,957 |
| *a. Dependent Variable: MHC-SF sum* | | | | | | | | |

**Supporting information for manuscript:**

Mental well-being in Swedish adolescents 2014-2023: a repeated population-based cross-sectional study focusing on temporal variations and differences between groups (Lena Uvhagen, Johanna Gustafsson, Fredrik Söderqvist)

**S1 Table. Regression coefficients**
