# Supplementary material for: Mental well-being in Swedish adolescents 2014–2023: A repeated population-based cross-sectional study focusing on temporal variations and differences between groups
Source: PLoS One. 2025 May 28;20(5):e0323963. doi: 10.1371/journal.pone.0323963 (PMC12118832; doi:10.1371/journal.pone.0323963)
Supplement: Attachment — Submitted filename: Response to reviewers 250202.docx [file pone.0323963.s003.docx]

02/02/2025

PLOS ONE

Dear Editor and reviewer,

Thank you for reviewing the manuscript *Mental well-being in Swedish adolescents 2014-2023: a repeated population-based cross-sectional study focusing on temporal variations and differences between groups*, and for considering it for publication in PLOS ONE. Below, we have responded to each point raised by editor and reviewer as follows:

| **Editor & Reviewer comments #** | **Revision** | **Line ^[1]*^** |
| --- | --- | --- |
| 1. Please ensure that your manuscript meets PLOS ONE's style requirements, including those for file naming. | The manuscript has been revised to ensure that it meets PLOS ONE´s style requirements. |  |
| 2. Please provide additional details regarding participant consent. | Clarifications regarding the participant consent have been made in the Materials and methods section in the manuscript and in the online submission information. The clarifications mainly concern the information that the participants received before the study. | 115-121, 127-128 |
| 3a. If there are ethical or legal restrictions on sharing a de-identified data set, please explain them in detail. | The Data Availability Statement have been updated and clarified according to point 3a. The clarifications states that data can be shared upon request to corresponding author, that public sharing is not possible due to ethical and legal restrictions concerning the student's consent and data ownership, and that the data is owned by a third party who has restrictions. |  |
| 4. Data availability. | Please see point 3a above. |  |
| 5. Please review your reference list to ensure that it is complete and correct. | The reference list has been revised to ensure that it is complete and correct. This includes the adding of one reference (#34) as a result of the new paragraph on demographics raised by reviewer, see below. |  |
| **Additional editor and reviewer comments:** | **Revision** | **Line** |
| Justifications for choice of study design and choice of statistical analyses | Regarding study design, we have clarified the choice of study design to fulfil the overall aim in the first paragraph in the *Study design, population and data collection* section. The headline has been changed to better match the content of the section.  Regarding choice of statistical analyses, the reasons for choosing Reverse Helmert’s contrast is clarified.  We have also added some information on the assumption underlying the reliability of the performed regression analysis.  In addition to these clarifications, we consider the section *Statistical analysis* to include a relatively detailed description of each step of the analysis and how it relates to each research questions and hence why it is justified. | 100-102  201-202  216-217 |
| A description of the demographics in Västmanland County is of value also for the discussion of generalization of the findings | Demographics of Västmanland county has been added in the *Study design, population and data collection* section in the manuscript. In the headline of this section, “participants” has been replaced with “population” to include the context.  Additionally, the generalisability of the findings has also been commented on in the *Discussion* section. | 105-110  331-334 |
| Description of the reliability and validity of the measures MHC-SF and SSES in the section of material and methods instead of in the discussion | Description of validity and reliability of the measures of Mental well-being and Self-reported socioeconomic status has been elaborated and moved from the Discussion to the Material and methods section. | 141-144, 170-172 |
| In the result section is missing data described, how was it treated? | The handling of missing data has been described in the Results section. | 230-234 |

We hope that these revisions are sufficient and fulfil your expectations. In addition to these revisions raised by editor and reviewer, we also made some copyediting changes after receiving go-ahead from editor via e-mail 24/01/2025. These changes does not include factual changes, only language editing, and have been made mainly in the discussion but also the last paragraph of the abstract, the conclusions and one paragraph in the introduction. To increase readability of the manuscript, these changes are not made with marked changes.

If there are any remaining revision needs or questions, please do not hesitate to contact me.

Yours sincerely,

Lena Uvhagen

PhD Student,

University Health Care Research Centre, Faculty of Medicine and Health, Örebro University

Email: lena.uvhagen@oru.se

Phone: +46 19 602 75 68, Mobile: +46 72 143 22 85

1. ^*^ In the manuscript version with tracked changes
